# Supplementary material for: The Presence of the Temporal Horn Exacerbates the Vulnerability of Hippocampus During Head Impacts
Source: Front Bioeng Biotechnol. 2022 Mar 22;10:754344. doi: 10.3389/fbioe.2022.754344 (PMC8980591; doi:10.3389/fbioe.2022.754344)
Supplement: Supplementary file 1 [file DataSheet1.docx]

**Appendix A: Development of finite element head model without the temporal horn**

The finite element (FE) head model without the temporal horn (i.e., the no-temporal-horn (NTH)-Model) used in this study was previously established at KTH Royal Institute of Technology in Stockholm, Sweden (Zhou et al., 2020). The geometry of the head model was extracted from an averaged magnetic resonance imaging (MRI) head template database (Fillmore et al., 2015). High-resolution T1- and T2-weighted images were segmented using the Freesurfer 7 (Fischl, 2012). The segmentation was subsequently processed by the 3D Slicer (Pieper et al., 2004) to obtain the surfaces of the skull, the brain, the third and the lateral ventricles, with the temporal horn being disregarded. All surfaces then served as an input to the Hexotic software, generating all hexahedron elements based on an octree algorithm (Maréchal, 2009). The falx and tentorium, which were almost invisible in the MRI, were manually created as shell elements based on the anatomical illustrations, while the pia mater and dura mater were generated by finding the outer faces of brain elements and subarachnoid cerebrospinal fluid (CSF) elements, respectively.

The material representation of simulated head components is summarized in Table A1 and Table A2. For the brain, a second-order Ogden-based hyperelastic constitutive law was used to describe the nonlinear behavior of the brain tissue, with additional linear viscoelastic terms to account for rate dependence. The subarachnoid CSF was modeled as a nearly incompressible material and shared interfacial nodes with the brain and skull. Mechanical properties of the intracranial membrane (i.e., pia mater and falx/tentorium/dura mater) were determined from the averaged material stress-strain curves from the tissue experiments. The brain, subarachnoid CSF, and intracranial membranes were modeled as Lagrangian elements (Zhou et al., 2020). In particular, the ventricles were represented by arbitrary Lagrange-Eulerian (ALE) fluid elements, and their responses were coupled to the brain via a fluid-structure interaction (FSI) scheme, which is detailed in the “Cerebral ventricle modeling” and “Brain-ventricle interface modeling” sections of the current study. Note that, in the NTH-model, the temporal horn was substituted as brain parenchyma with the material constants in Table A2.

Table A1. Material properties for the finite element head model. K is Bulk modulus and N/A is not applicable.

| Tissue | Young's modulus (MPa) | Density (kg/dm3) | Poisson's ratio | Reference |
| --- | --- | --- | --- | --- |
| Cortical bone | 15000 | 2.00 | 0.22 | (Kleiven, 2007) |
| Porous bone | 1000 | 1.3 | 0.24 | (Kleiven, 2007) |
| Brain | Hyper-Viscoelastic  (Table A2) | 1.04 | 0.5 | (Kleiven, 2007) |
| CSF/Ventricle | K = 2.1 GPa | 1.00 | N/A | (Kleiven, 2007) |
| Dura/Falx/Tentorium | Average stress-strain curve | 1.13 | N/A | (Aimedieu and Grebe, 2004) |
| Pia | Average stress-strain curve | 1.13 | N/A | (Van Noort et al., 1981) |

Table A2. Ogden hyperelastic and liner viscoelastic constants for the brain material modeling. μ_i_ and α_i_ are Ogden parameters, G_i_ represents the 6 shear relaxation moduli, β_i_ are the 6 decay constants.

| Parameter | Value | Parameter | Value |
| --- | --- | --- | --- |
| μ_1_ (Pa) | 53.8 | α_1_ | 10.1 |
| μ_2_ (Pa) | -120.4 | α_2_ | -12.9 |
| G_1_ (MPa) | 0.32 | β_1_ (s^-1^) | 10^6^ |
| G_2_ (kPa) | 78 | β_2_ (s^-1^) | 10^5^ |
| G_3_ (kPa) | 6.2 | β_3_ (s^-1^) | 10^4^ |
| G_4_ (kPa) | 8.0 | β_4_ (s^-1^) | 10^3^ |
| G_5_ (kPa) | 1.0 | β_5_ (s^-1^) | 10^2^ |
| G_6_ (kPa) | 3.0 | β_6_ (s^-1^) | 10^1^ |

**Appendix B: Validation of brain-skull relative motion and brain strain**

The strain response and brain-skull relative motion estimated by the model with the temporal horn (i.e., the TH-Model) was validated against the available experimental data presented by Hardy et al. (2007) and Zhou et al. (2019). In Hardy et al. (2007), a high-speed, biplane X-ray system was used to track the motion of the radiopaque neutral density targets (NDTs) implanted in cluster array within the cadaveric brain. The NDT initial coordinates and motion were obtained with respect to an anatomical coordinate system with the c.g. of the head being the origin. Strain in the volume encompassed by the NDT cluster was calculated by imposing the experimentally measured NDT motions to an auxiliary model that was developed by connecting each NDT to its neighboring counterparts to form tetra elements (Zhou et al., 2019).

In the present work, three representative cases are selected, including C288-T3 (sagittal impact), C380-T1 (coronal impact), and C380-T2 (horizontal impact). To numerically reproduce the experimental impacts, the recorded head kinematic curves were imposed to the node which locates at the center of gravity of the corresponding cadaveric head and is rigidly attached to the skull. To approximate the specimen anthropometry, the model was scaled independently in directions of both the depth and breadth to match the reported cadaveric head sizes. The node nearest to the start position of an experimental NDT target was taken as the marker location in the model. Motions of the identified nodes with respect to the skull along three anatomical coordinate directions are obtained from the whole head model simulation with the detailed results presented in **Fig. B1-B3**. Following the procedures established by Zhou et al. (2019), the initial positions of the identified nodes and the nodal motion responses predicted in the model was used to calculate the strain responses, specifically first principal Green-Lagrange strain and shear Green-Lagrange strain, of the brain model. The strain validation results are shown in **Fig. B4.**

**Fig B1. Comparison between experimental and simulated brain-skull relative motion for the experiment C288-T3.**

**Fig B1. Comparison between experimental and simulated brain-skull relative motion for the experiment C380-T1.**

Fig B3. Comparison between experimental and simulated brain-skull relative motion for the experiment C380-T2.

Fig B4. Comparison between experimental and simulated brain strains.

**Appendix C: Loading curves for 3 concussive impacts and 3 sub-concussive impacts**

**Case 1**

**Case 3**

**Case 2**

**Case 4**

**Case 6**

**Case 5**

Fig C1. Loading conditions for 3 concussive impacts (Cases 1-3) and 3 sub-concussive impacts (Cases 4-6). The X, Y, and Z axes are the same as those in the skull-fixed coordinate system in Fig. 1a.

**Appendix D: Volume ratio of the hippocampal subfields and whole hippocampus with strain over 0.2 and strain rate over 30 s^-1^**

Case 1

Case 2

Case 3

**Volume ratio of strain over 0.2**

**(Concussive cases)**

**Volume ratio of strain over 0.2**

**(Sub-concussive cases)**

**A**

**B**

Case 4

Case 5

Case 6

**Volume ratio of strain rate over 30 s^-1^**

**(Concussive cases)**

**Volume ratio of strain rate over 30 s^-1^**

**(Sub-concussive cases)**

**C**

**D**

**Hippocampus**

Volume ratio

Volume ratio

Volume ratio

Case 1

Case 2

Case 3

Case 4

Case 5

Case 6

Volume ratio

Volume ratio

Volume ratio

**Fig. D1.** **Volume ratio of the maximum principle strain over 0.2 and strain rate over 30 s^-1^ in the hippocampal subfields and the whole hippocampus between the TH-Model and NTH-model of 3 concussive impacts (Cases 1-3) and 3 sub-concussive impacts (Cases 4-6).**

**Appendix E: Comparison of strain and strain rate in the non-hippocampal regions**

**Non-hippocampal regions**

Case 1

Case 2

Case 3

**Strain**

**(Concussive cases)**

**Strain**

**(Sub-concussive cases)**

**A**

**B**

Case 4

Case 5

Case 6

Case 1

Case 2

Case 3

Case 4

Case 5

Case 6

**Strain rate**

**(Concussive cases)**

**Strain rate**

**(Sub-concussive cases)**

**C**

**D**

Strain

Strain

Strain

Strain rate (s^-1^)

Strain rate (s^-1^)

Strain rate (s^-1^)

**Fig E1. Comparison of strain and strain rate in the non-hippocampal regions between the TH-model and NTH-model of 3 concussive impacts (Cases 1-3) and 3 sub-concussive impacts (Cases 4-6).** (A) Comparison of strain in the non-hippocampal periventricular regions of 3 concussive impacts. (B) Comparison of strain in the non-hippocampal periventricular regions of 3 sub-concussive impacts. (C) Comparison of strain rate in the non-hippocampal periventricular regions of 3 concussive impacts. (D) Comparison of strain rate in the non-hippocampal periventricular regions of 3 sub-concussive impacts. Percentages in strain difference and strain rate difference are calculated with the results of the NTH-model as the baseline. Ventral DC: ventral diencephalon; CC: Corpus callosum.

Aimedieu, P., and Grebe, R. (2004). Tensile strength of cranial pia mater: preliminary results. *Journal of neurosurgery* 100**,** 111-114.

Fillmore, P.T., Phillips-Meek, M.C., and Richards, J.E. (2015). Age-specific MRI brain and head templates for healthy adults from 20 through 89 years of age. *Frontiers in aging neuroscience* 7**,** 44.

Fischl, B. (2012). FreeSurfer. *Neuroimage* 62**,** 774-781.

Hardy, W.N., Mason, M.J., Foster, C.D., Shah, C.S., Kopacz, J.M., Yang, K.H., King, A.I., Bishop, J., Bey, M., and Anderst, W. (2007). A Study of the Response of the Human Cadaver Head to Impact. *Stapp Car Crash J* 51**,** 17-80.

Kleiven, S. (2007). Predictors for traumatic brain injuries evaluated through accident reconstructions. *Stapp car crash journal* 51**,** 81-114.

Maréchal, L. (2009). "Advances in octree-based all-hexahedral mesh generation: handling sharp features," in *Proceedings of the 18th international meshing roundtable*. Springer), 65-84.

Pieper, S., Halle, M., and Kikinis, R. (Year). "3D Slicer", in: *2004 2nd IEEE international symposium on biomedical imaging: nano to macro (IEEE Cat No. 04EX821)*: IEEE), 632-635.

Van Noort, R., Black, M., Martin, T., and Meanley, S. (1981). A study of the uniaxial mechanical properties of human dura mater preserved in glycerol. *Biomaterials* 2**,** 41-45.

Zhou, Z., Li, X., and Kleiven, S. (2020). Biomechanics of periventricular injury. *Journal of neurotrauma* 37**,** 1074-1090.

Zhou, Z., Li, X., Kleiven, S., and Hardy, W. (2019). Brain Strain from Motion of Sparse Markers. *Stapp car Crash Journal* 63**,** 1-27.
